# Genetic basis of myeloid transformation in familial platelet disorder/acute myeloid leukemia patients with haploinsufficient *RUNX1* allele

**DOI:** 10.1038/bcj.2015.81

**Published:** 2016-02-05

**Authors:** M Sakurai, H Kasahara, K Yoshida, A Yoshimi, H Kunimoto, N Watanabe, Y Shiraishi, K Chiba, H Tanaka, Y Harada, H Harada, T Kawakita, M Kurokawa, S Miyano, S Takahashi, S Ogawa, S Okamoto, H Nakajima

**Affiliations:** 1 Division of Hematology, Department of Internal Medicine, Keio University School of Medicine, Tokyo, Japan; 2 Department of Pathology and Tumor Biology, Graduate School of Medicine, Kyoto University, Kyoto, Japan; 3 Department of Hematology and Oncology, Graduate School of Medicine, The University of Tokyo, Tokyo, Japan; 4 Department of Transfusion Medicine & Cell Therapy, Keio University School of Medicine, Tokyo, Japan; 5 Laboratory of DNA Information Analysis, Human Genome Center, The Institute of Medical Science, The University of Tokyo, Tokyo, Japan; 6 Laboratory of Sequence Analysis, Human Genome Center, The Institute of Medical Science, The University of Tokyo, Tokyo, Japan; 7 Department of Hematology, Juntendo University School of Medicine, Tokyo, Japan; 8 Department of Hematology/Oncology, The Institute of Medical Science, The University of Tokyo, Tokyo, Japan

Familial platelet disorder/acute myeloid leukemia (FPD/AML) is an autosomal dominant inherited disorder characterized by thrombocytopenia and high propensity to various hematological malignancies. FPD/AML is caused by monoallelic mutations of *RUNX1*, which are in many cases point mutations disrupting DNA-binding or transactivating capacities of RUNX1, and these mutations are considered to act in dominant-negative manner to various degrees for residual wild-type allele.^1, 2^ Interestingly, some FPD/AML traits present monoallelic loss of *RUNX1* gene by microdeletion of chromosome 21.^1^ In such cases, no mutations were found in the residual *RUNX1* allele, thus suggesting that haploinsufficiency of *RUNX1* is sufficient to cause FPD/AML.

Genetic basis of leukemic transformation in FPD/AML patients still remains elusive. We have recently reported recurrent mutation of *CDC25C* in FPD/AML patients, and suggested that this mutation is one of the early events, which defines pre-leukemic state.^3^ However, all pedigrees that we examined carried point mutations of *RUNX1*, and no cases with monoallelic *RUNX1* loss were present. We suspected that the dosage of *RUNX1* activity may affect the transforming processes, and FPD/AML with haploinsufficient *RUNX1* allele might require unique genetic events for transformation that are distinct from cases with *RUNX1* point mutation. In order to identify collaborating mutations with haploinsufficient *RUNX1* allele, we performed genome-wide mutational analyses of two transformed cases of FPD/AML with monoallelic *RUNX1* loss. This study was conducted with approval from the internal review board of Keio University School of Medicine and conformed to the principles outlined in the Declaration of Helsinki for the use of human tissue or subjects. Samples from the patients were collected with written informed consent.

## Patient 1

**Figure 1:**
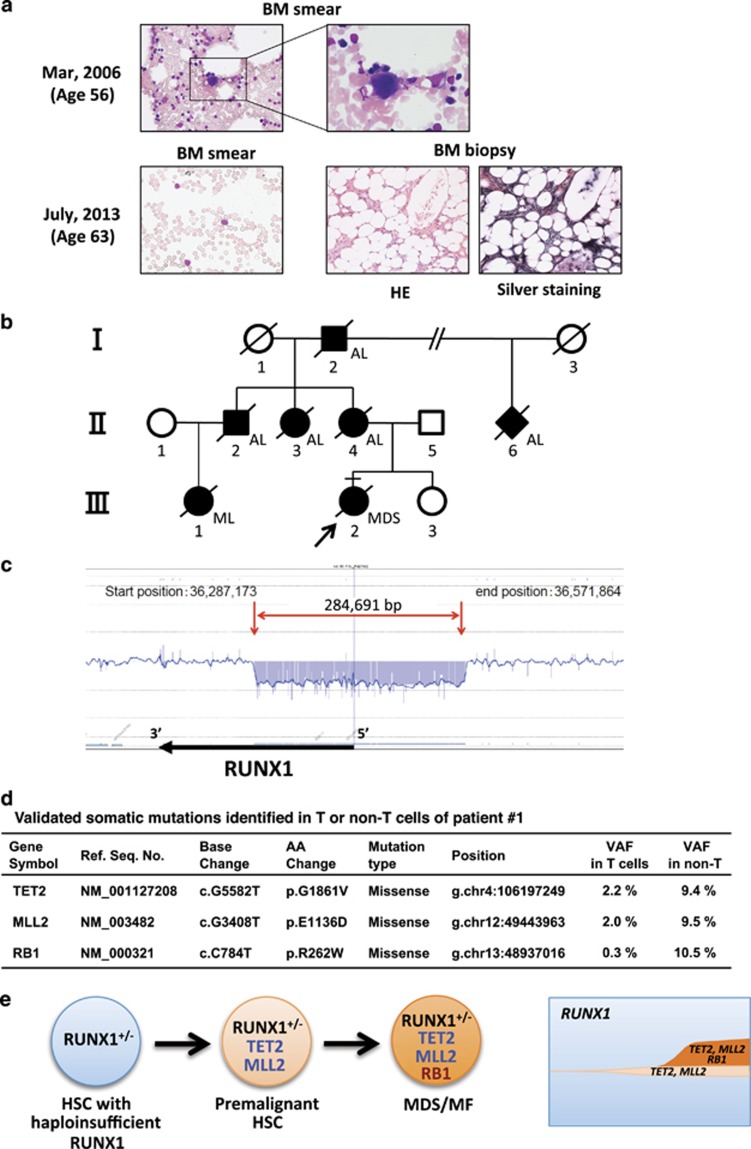
Clinical, pathological and molecular data of patient 1. (**a**) Photographs of BM smears and biopsies. BM smears were stained with May-Grunwald Giemsa staining. BM biopsies were stained with Hematoxylin-Eosin (HE) or silver staining. Original magnification; 400 × or 1000 × . (**b**) Family tree of the pedigree. Filled symbols; affected members, slashed symbols; deceased members, arrow; proband, square; male, circle; female, diamond; sex not determined. AL; acute leukemia, ML; malignant lymphoma. (**c**) Schematic data of array-comparative genomic hybridization (aCGH) on *RUNX1* locus. aCGH was performed using custom-made oligonucleotide microarrays (Agilent Technologies, Santa Clara, CA, USA) covering human chromosome 21, including *RUNX1* locus with resolution of several hundred base pairs. A chromosomal region of heterozygous microdeletion is indicated by red arrows. (**d**) Validated somatic mutations identified in T or non T cells of patient 1. Whole-exome sequencing was performed as described previously.^13^ Whole-exome capture was accomplished with the cDNA library prepared by SureSelect Human All Exon V5 (Agilent Technologies). Captured targets were subjected to massively parallel sequencing by Illumina HiSeq 2000 (San Diego, CA, USA). Candidate somatic nucleotide variants were validated by deep sequencing. (**e**) A proposed model of disease progression and clonal architecture of patient 1. HSCs with haploinsufficient *RUNX1* first acquire mutations of *TET2* and *MLL2*, which establishes pre-leukemic state. Premalignant HSCs then acquire RB1 mutation to progress into full-blown myelodysplastic syndrome with myelofibrosis (MF).

The patient was referred to our hospital for persisting thrombocytopenia from childhood. Bone marrow (BM) examination at the age of 56 revealed normocellular marrow with micromegakaryocytes (Figure 1a), which led to a diagnosis of idiopathic thrombocytopenic purpura. However, she started to develop progressive pancytopenia at the age of 63, when the marrow presented severe hypoplasia with moderate fibrosis and no blast proliferation (Figure 1a). Of note, dysplasia was not evident and the karyotype was normal.

Family history revealed high penetrance of hematological malignancies such as AML and myelodysplastic syndrome, which suggested an inherited mutation of *RUNX1* (Figure 1b). However, no mutations were discovered in the coding region of *RUNX1*, which suggested the chromosomal microdeletion encompassing *RUNX1* locus, as was reported in some cases of FPD/AML.^4^ Indeed, array-comparative genomic hybridization analysis of the patient's somatic DNA revealed ~285 kb heterozygous deletion including the promoter and the 5′-half of *RUNX1* gene (Figure 1c). These data clearly demonstrated that this pedigree was an FPD/AML with haploinsufficient *RUNX1* allele.

We tried to dissect the genetic basis underlying BM failure by whole-exome sequencing of genomic DNA extracted from CD3^+^ T cells and CD3^−^ non T cells of patient's peripheral blood (Figure 1d). This revealed mutations of *TET2* and *MLL2* genes in peripheral T cells at low variant allele frequency (2.0–2.2%). Interestingly, non T cells harbored mutation of *RB1* gene in addition to *TET2* and *MLL2* at variant allele frequency of ~10%. These data suggest that *RUNX1*-haploinsufficient hematopoietic stem cells acquired mutations in *TET2* and *MLL2* genes to establish premalignant hematopoietic stem cells, which then underwent malignant transformation by acquiring *RB1* mutation (Figure 1e).

## Patient 2

**Figure 2:**
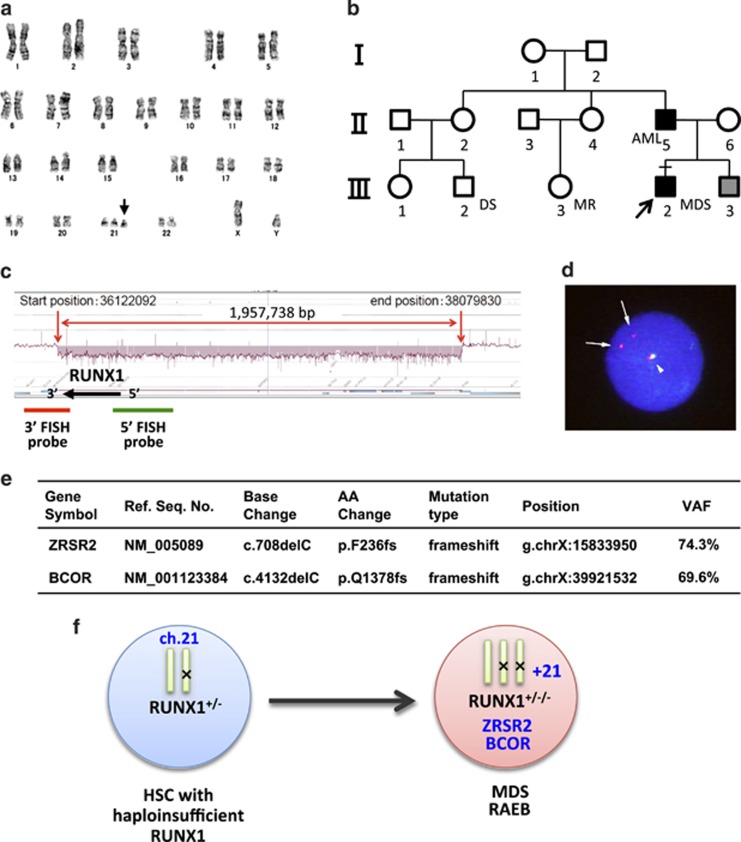
Clinical, cytogenetic and molecular data of patient 2. (**a**) Karyotype analysis of BM cells. Arrow indicates additional chromosome 21. (**b**) Family tree of the pedigree. Filled symbols; affected members, gray symbols; members with thrombocytopenia, arrow; proband, square; male, circle; female. DS; Down syndrome-like phenotype, MR; mental retardation. (**c**) Schematic data of aCGH on *RUNX1* locus. aCGH was performed as described in Figure 1. A chromosomal region of heterozygous microdeletion is indicated by red arrows. FISH probes for 5′- or 3′-*RUNX1* locus are indicated by green or red lines, respectively. (**d**) FISH analysis of BM cells for *RUNX1* locus. 99.7% (997/1000) of evaluable cells presented trisomy 21 with abnormal signals for *RUNX1* locus. Arrows indicate abnormal chromosome 21 hybridized only with 3′-probe (red signals). Arrowhead indicates normal chromosome 21 hybridized to both 5′- and 3′-probes. (**e**) Validated somatic mutations identified in BMMNCs of patient 2. Whole-exome and deep sequencing was performed as described in Figure 1. (**f**) A proposed model of disease progression of patient 2. In transformed cells, abnormal chromosome 21 with haploinsufficient *RUNX1* allele duplicated, which collaborated with mutations of *ZRSR2* and *BCOR* genes to initiate myelodysplastic syndrome.

The patient presented thrombocytopenia (46 × 10^9^/l) and was diagnosed with lower-risk myelodysplastic syndrome by BM examination at the age of 29. Two years later, he presented progressive leukocytopenia (2.0 × 10^9^/l) with recurrent bacterial infection. BM aspirates revealed 5.6% of blasts with abnormal karyotype, including trisomy 21 (Figure 2a), which led to a diagnosis of myelodysplastic syndrome: RAEB-1. BM blasts further increased to 13.0% in the following 2 years, and stem cell transplantation was subsequently performed using human leukocyte antigen-matched cord blood as a donor.

Family history showed that his father developed AML and his younger brother presented thrombocytopenia (Figure 2b). Interestingly, two cousins of the proband presented mental retardation and Down syndrome-like phenotype, respectively. High incidence of hematological malignancies and metal retardation suggested a rare type of FPD/AML caused by microdeletion of chromosome 21, as reported previously.^5^ Indeed, array-comparative genomic hybridization analysis using the patient's somatic DNA confirmed ~2 Mb heterozygous deletion in chromosome 21 encompassing the entire *RUNX1* gene and a large genomic region of 5′-*RUNX1* (Figure 2c), which indicates that this pedigree is an FPD/AML with haploinsufficient *RUNX1* allele.

We next investigated the genetic events critical for myeloid transformation in this patient. As described above, trisomy 21 was noted in the BM cells, which was considered to have contributed to leukemic transformation. Interestingly, fluorescent *in situ* hybridization analysis of the BM cells revealed duplication of ‘abnormal' chromosome 21 lacking hybridization to the 5′- probe for *RUNX1* locus (Figures 2c and d). These data strongly suggested that maintaining haploinsufficient *RUNX1* allele with trisomy 21 was critical for transformation. Furthermore, mutations of *ZRSR2* and *BCOR* genes were detected by whole-exome sequence of genomic DNA from BM mononuclear cells (Figure 2e). These data suggest that disruption of *ZRSR2* and *BCOR* genes combined with duplication of abnormal chromosome 21 with *RUNX1* deletion is critical for myeloid transformation (Figure 2f).

We have recently reported recurrent *CDC25C* mutation in ~50% of FPD/AML patients. Hierarchical architecture analysis showed that *CDC25C* mutation was an early event during transformation, which defines a pre-leukemic clone.^3^ Mutant CDC25C was shown to confer cells with a proliferative advantage by facilitating their mitotic entry. In the present cases, however, *CDC25C* mutation was absent, and instead, *TET2* mutation or trisomy 21 was identified in each patient. Intriguingly, both of these genetic alterations confer cells with clonal advantage similarly as *CDC25C* mutation. *TET2* mutation has been shown to augment stem cell capacity of hematopoietic stem cells, which then facilitate the expansion of mutated clones.^6, 7, 8^ Trisomy 21 is a well-known chromosomal abnormality associated with AML,^9, 10^ and it was shown to confer hematopoietic progenitors with enhanced self-renewal capacity without inducing leukemia.^4, 11^ Interestingly, trisomy 21 has been reported in some transformed or non-transformed cases of FPD/AML.^4, 12^ In these cases including ours, duplication occurred invariably with abnormal chromosome 21 carrying *RUNX1* mutation, maintaining a single copy of wild-type *RUNX1* gene unaffected. This implies that amplification of dose sensitive genes on chromosome 21 combined with *RUNX1* haploinsufficiency is a critical step for leukemic transformation in FPD/AML.

Taken together, our study suggests that *RUNX1* haploinsufficiency collaborates with genetic alterations conferring clonal advantage such as *TET2* mutation or trisomy 21 to establish pre-leukemic state, similarly as *RUNX1* point mutation does with *CDC25C* mutation. This study adds valuable molecular insight into the transforming processes in FPD/AML patients.
